# Development and application of quadruplex real time quantitative PCR method for differentiation of Muscovy duck parvovirus, Goose parvovirus, Duck circovirus, and Duck adenovirus 3

**DOI:** 10.3389/fcimb.2024.1448480

**Published:** 2024-08-19

**Authors:** Haojie Wang, Jianxing Chen, Tongqing An, Hongyan Chen, Yue Wang, Liangquan Zhu, Changqing Yu, Changyou Xia, He Zhang

**Affiliations:** ^1^ State Key Laboratory for Animal Disease Control and Prevention, Harbin Veterinary Research Institute, Chinese Academy of Agricultural Sciences, Harbin, China; ^2^ China Institute of Veterinary Drug Control, Beijing, China; ^3^ School of Advanced Agricultural Sciences, Yibin Vocational and Technical College, Yibin, China

**Keywords:** Muscovy duck parvovirus (MDPV), Goose parvovirus (GPV), Duck circovirus (DuCV), Duck adenovirus 3 (DAdV-3), quadruplex, qPCR

## Abstract

**Introduction:**

Muscovy duck parvovirus (MDPV), Goose parvovirus (GPV), Duck circovirus, (DuCV) and Duck adenovirus 3 (DAdV-3) are important pathogens that cause high morbidity and mortality in ducks, causing huge economic loss for the duck industry.

**Methods:**

The present study, a quadruplex one-step real time quantitative PCR method for the detection of MDPV, GPV, DuCV, and DAdV-3 was developed.

**Results:**

The results showed that assay had no cross-reactivity with other poultry pathogens [Duck plague virus (DPV), Duck tembusu virus (DTMUV), H6 avian influenza virus (H6 AIV), New duck reovirus (NDRV), Newcastle disease virus (NDV), H4 avian influenza virus (H4 AIV), *Escherichia coli* (*E. coli*), Muscovy duck reovirus (MDRV), Egg drop syndrome virus (EDSV), *Pasteurella multocida* (*P. multocida*)]. The sensitivity result showed that the limits of detection for MDPV, GPV, DuCV, and DAdV-3 were 10, 10, 1 and 10 copies/µl, respectively; The coefficients of variation intra- and inter-method was 1-2%; The range of linear (109 to 103 copies/µL) demonstrated the R2 values for MDPV, GPV, DuCV, and DAdV-3 as 0.9975, 0.998, 0.9964, and 0.996, respectively. The quadruplex real time quantitative PCR method efficiency was 90.30%, 101.10%, 90.72%, and 90.57% for MDPV, GPV, DuCV, and DAdV-3, respectively. 396 clinical specimens collected in some duck sausages from June 2022 to July 2023 were simultaneously detected using the established quadruplex real time quantitative PCR method and the reported assays. The detection rates for MDPV, GPV, DuCV, and DAdV-3 were 8.33% (33/396), 17.93% (71/396), 33.58% (133/396), and 29.04% (115/396), respectively. The agreement between these assays was greater than 99.56%.

**Discussion:**

The developed quadruplex real-time quantitative PCR assay can accurately detect these four viruses infecting ducks, providing a rapid, sensitive, specific and accurate technique for clinical testing.

## 1 Introduction

With the rapid growth of duck farming in China, the density of duck flocks has increased and a variety of infectious diseases endanger the health of the ducks (Shi et al., 2022). Among the different pathogens, Muscovy duck parvovirus (MDPV), Goose parvovirus (GPV), Duck circovirus (DuCV) and Duck adenovirus 3 (DAdV-3) can cause similar symptoms of slow growth, diarrhoea, enteritis and liver haemorrhages in ducks, which are difficult to distinguish (He et al., 2022; Li et al., 2022; Shi et al., 2022; Huo et al., 2023). In particular, duck circovirus tends to cause mixed infections or secondary infections, leading to aggravation of the disease. Infection with these four viruses is causing serious economic losses in the duck farming industry (Liao et al., 2022; Wang et al., 2023b; Yin et al., 2023).

The first report of MDPV was in France in 1989 (Lin et al., 2019), and the first case of the virus was in China in the 1990s (He et al., 2022). GPV was reported in China in the early 1960s (Liu et al., 2023). MDPV and GPV are both waterfowl belonging to the *Aveparvovirus*, *Dependoparvovirus*, a single-stranded DNA genome of about 5100 bp in length (Wang et al., 2021). These viruses have two inverted terminal repeats (ITRs) at the 5’ and 3’ ends, forming a hairpin structure. Includes two major open reading frames (ORFs) (Zhao et al., 2014; Wang et al., 2021). The left ORF that encodes for non-structural proteins (NS1 and NS2), which is participated in the replication and regulation of viruses and are also known as regulatory proteins (REPs) (Yu et al., 2016; Wan et al., 2018c). The three capsid proteins (VP1, VP2 and VP3) encoded by the right ORF, which plays an important role in the tropism, pathogenicity and host range of the virus (Li et al., 2021). There is a high degree of homology between MDPV and GPV, but there are significant differences in the antibody neutralisation tests and the host range (Wan et al., 2018c). Both MDPV and GPV are pathogenic to ducks (Wan et al., 2018c). In recent years, the prevalence of MDPV and GPV in duck flocks has been high (33.33% and 25%) and mixed infections have been severe (8.33%) (Wan et al., 2018c). Therefore, it is particularly important to distinguish between MDPV and GPV infected ducks.

Duck circovirus (DuCV) was first Covered in Germany in 2003, detected in Taiwan and mainland China in 2006 and 2008 respectively (Hattermann et al., 2003; Wan et al., 2011), and is now increasingly prevalent in major duck breeding areas around the world (Ji et al., 2020; Tran et al., 2022). The prevalence of DuCV was 43.09% in Yunnan duck flock in 2018-2019 (Liu et al., 2020a). DuCV is a circular single-stranded DNA virus, about 2 kb in length, including two ORFs, ORF1 encoding the replicase (V1) and ORF2 encoding the capsid (C1) protein (Huang et al., 2023). There are two non-coding intergenic regions (IRs) between the 5′and 3′ends of the two major ORFs (Liao et al., 2022). DuCV can infect ducks of all breeds and ages, and can be found in ducks at any time of the year. In addition, DuCV infection leads to herd immunosuppression, predisposing to the occurrence of secondary infections with pathogens such as GPV, MDPV, DAdV-3 and FAdV-4 (Liu et al., 2020b; Shen et al., 2023).

Duck adenovirus 3 (DAdV-3) is a new type of DAdV that has been discovered in recent years, which is highly pathogenic, and the clinical autopsy lesions of infected ducks mainly showed enlargement, haemorrhage and necrosis of the liver and kidney (Shi et al., 2022). DAdV-3 has the general characteristics of adenoviruses, spherical viral particles without a vesicular membrane, icosahedral symmetry and double-stranded DNA of 43,842 bp (Tan et al., 2024). DAdV-3 has one more fibril (Fiber-2), than DAdV-1and DAdV-2 (Tan et al., 2024). The Fiber-2 gene has high type-specific and subgeneric specificity, which is related to the virulence of DAdV-3, and is an important protective immunogen that has been used as a diagnostic target (Chen et al., 2019; Shao et al., 2022; Shi et al., 2022).

In summary, MDPV, GPV, DuCV and DAdV-3 are commonly found in duck flocks, with similar clinical signs, pathological changes and susceptibility to mixed infections leading to greater damage in duck flocks. Therefore, a sensitive and specific method must be developed to detect these pathogens. Fluorescence quantitative PCR method uses specific primers and probes to amplify the target segments with good accuracy (Meng et al., 2023). Meanwhile, the target gene is dual controlled by primers and probes with highly specific (Wang et al., 2023a). Compared to the traditional PCR, fluorescent quantitative method has the merits of higher sensitivity, high throughput and direct quantification (Xin et al., 2023). There are no reported assays for the simultaneous diagnosis of MDPV, GPV, DuCV and DAdV-3. Therefore, we selected four DNA viruses, MDPV, GPV, DuCV and DAdV-3, without the need for reverse transcription, and established a quadruplex real time quantitative PCR method to provide technical service for clinical defence and control of these pathogens.

## 2 Materials and methods

### 2.1 Bacterials, viruses, and clinical specimens

MDPV, GPV, DuCV, DAdV-3, *Escherichia coli* (*E. coli*), Duck tembusu virus (DTMUV), *Pasteurella multocida* (*P. multocida*) were kept in our laboratory. H6 avian influenza virus (H6 AIV), New duck reovirus (NDRV), Newcastle disease virus (NDV), Muscovy duck reovirus (MDRV), Duck plague virus (DPV), Egg drop syndrome virus (EDSV), H4 avian influenza virus (H4 AIV) were supplied from China institute of Veterinary Drug Control. 396 clinical specimens of ducks (blood, lymph nodes, spleens, renal, intestinal, and et al) with open-mouth respiration, panting, lethargy, refusal to eat, crouching, and dishevelled plumage were collected from some duck sausages, from August 2022 to September 2023. 396 Clinical samples are stored at -80°C.

### 2.2 Extraction of genome

The bacterial DNA was extracted using the FastPure Enhanced EndoFree Plasmid Maxi (Plus) Kit (Vazyme, China), and viral nucleic acids were extracted using the *MagicPure* ^®^ Simple Viral DNA/RNA Kit (TransGen Biotech, China). We used a NanoPhotometer^®^ (Thermo Fisher, USA) to test nucleic acids for concentration and purity and selected A260/A280 at 1.8~2.0 for storage and use at -20°C.

### 2.3 Primers and probes

Genome sequences of MDPV *ns* gene, GPV *ns* gene, DuCV *rep* gene and DAdV-3 *fiber-2* sequences were acquired from NCBI (https://www.ncbi.nlm.nih.gov/), and aligned using Megalign software. Based on the alignments, primers and probes for MDPV, GPV, DuCV and DAdV-3 were designed using the Beacon Designer 8.14 software (**Table 1**). Primers and probes were Tsingke Biotechnology Co., Ltd. (Beijing, China).

**Table 1:** Primers and probes of MDPV, GPV, DuCV, DAdV-3.

Pathogen	Gene	Primers and probes	Sequences (5′ end to 3′ end)	length	Accession no.
MDPV	*NS*	F	TGCATCTCCCCATGGTTACTC	85 bp	MT450871.1
		R	TCCGTTTCGTCCTGATTGAAT		
		probe	FAM-CAGACAAAATCAAGAACAT-MGB		
GPV	*NS*	F	TCAAATGGGCCAACGACAAT	85 bp	MH444513.1
		R	ACGGGCCTTGGTAAGTTGGT		
		probe	Cy5-CAAAGTCCGAACGAATGA-MGB		
DuCV	*Rep*	F	TGCGCCAAAGAGTCGACATA	80 bp	MK814589.1
		R	GATGTCGCTTCYGCCAGATCA		
		probe	TAMRA-CCAGTCTCCAAGGGT-MGB		
DAdV-3	*fiber-2*	F	TCCGTTCTGGGAGCAAGCT	56 bp	KR135164.1
		R	CGGTGACAAACGGAGGATTT		
		probe	VIC-CGAGTGCCAACCCG-MGB		

### 2.4 Preparation of recombinant plasmid standards

The DNA of MDPV, GPV, DuCV, and DAdV-3 were used as a template to amplify the target fragment using the primers in **Table 2**, and then the amplification products were cleansed using Agarose Gel Purification and Recovery Kit (Biomed, China), and cloned to the *pEASY* ^®^-T1 Cloning vector. The ligation products were converted to *Trans1-T1 Phage Resistant Chemically Competent Cell* (TransGen Biotech, China). After being cultured for 12-16 h at 37°C. The positive strains were selected to expand the culture and the recombinant plasmid standards were acquired using FastPure EndoFree Plasmid Kit (Vazyme, China). The recombinant plasmid standards were named p-MAPV, p-GPV, p-DuCV, and p-DAdV-3, respectively. The concentrations determined by NanoPhotometer^®^ (Thermo Fisher, USA), select plasmids with A260/A280 at 1.8-2.0and then stored at -80°C. Each plasmid was diluted to 4×10^9^ copies/μL after conversion by the formula. The recombinant plasmid standards copy number was counted as follows:

**Table 2:** Primers used for recombinant plasmid standards construction.

Pathogens	Gene	Primers	Sequences (5′ end to 3′ end)	length	Accession no.
MDPV	*NS*	F	CACTTTCTAGGCCTCTGCAGATTT	349 bp	MT450871.1
		R	TGAGACATGTATCTCCCCAGAACA		
GPV	*NS*	F	ACCAGTAATACTGATATGTGTATG	357 bp	MH444513.1
		R	AATTGGAGCAACTGATGAGAACAA		
DuCV	*Rep*	F	AGTAACGCGCGAGCTGCCGCCCTT	423 bp	MK814589.1
		R	GTCATTTCCCGAGTAACCGTCCCA		
DAdV-3	*fiber-2*	F	CCAACAGATCAGACAATGGTGAAG	435 bp	KR135164.1
		R	ACATGGTTTCTGTATCATAGGCTA		

The recombinant plasmid standards copies/µL = (6.02×10^23^) × (X^*^ ng/µL × 10^−9^)/constructed plasmid length (bp) × 660(27).

* *X*: Standard plasmid concentration.

### 2.5 Optimising quadruplex real time quantitative PCR methods

The assay was performed using the ABI 7500 fast Real Time PCR system (Applied Biosystems, USA), and the reaction temperature and the concentration of primers and probes were optimised separately to obtain the optimal reaction conditions. The total reaction system was 25 µL. The experiment used 12.5 µL of 2 × Animal Detection U^+^ Probe Qpcr Super Premix (Vazyme, China); final concentrations of primers and probes were 0.1-1 µM; 2 µL of a mixture of the four pathogenic DNAs (10 ng/μl), as a template; 50 × ROX Reference Dye 2, and distilled water (ddH_2_O) to a total volume of 25 μL. The following parameters were used: 95°C for 30 s; 45 cycles of 95°C for 10 s and annealing and extension temperature (59°C, 60°C, 61°C, 62°C and 63°C) for 30 s. The optimal conditions were determined according to the minimum Ct values, the maximum Δ*Rn* and amplification curve.

### 2.6 Standard curve creation

The recombinant plasmid standards (p-MAPV, p-GPV, p-DuCV and p-DAdV-3) were mixed according to 1:1:1:1:1, and then diluted in 10-fold gradient, and the plasmids with the final reaction concentration of 1 × 10^9^ to 1 × 10^3^ copies/µl (3 replicates per concentration) were selected to establish the standard curves. At the end of the reaction, the standard curves were derived directly from the software. The correlation coefficient (R^2^), amplification efficiency (Eff%), and standard equation were counted.

### 2.7 Specificity verification

The nucleic acids of DTMUV, *E. coli*, *P. multocida*, NDRV, NDV, H4 AIV, H6 AIV, MDRV, DPV, EDSV were used as amplified templates to validate the specificity of the developed quadruplex real time quantitative PCR assay. The DNA of MDPV, GPV, DuCV, DAdV-3 were used as positive controls, and ddH_2_O were used as negative controls.

### 2.8 Sensitivity testing

The recombinant plasmid standards of p-MAPV, p-GPV, p-DuCV, and p-DAdV-3 were mixed (1:1:1:1), and then, the 10 times dilution from 1 × 10^9^ to 1 × 10^0^ copies/μL was used as amplified templates to the sensitivity of the quadruplex real time quantitative PCR method.

### 2.9 Reproducibility verification

The reproducibility of the established methods was assessed by performing intra- and inter-batch experiments. Four recombinant plasmid standards with different final concentrations (of a liquid) of 1 ×10^3^, 1 × 10^5^, and 1 ×10^7^ copies/µL were used as templates. All responses were replicated three times. The coefficient of variation (CVs) was counted to assess the reproducibility of the assay.

### 2.10 Clinical sample testing

396 Clinical specimens of ducks (blood, lymph nodes, spleens, renal, intestinal, and et al) were collected from some duck sausages, from August 2022 to September 2023. Tissue specimens were weighed 1.0 g; 600 μL saline was added, ground thoroughly, and the nucleic acids were acquired using the *MagicPure* ^®^ Simple Viral DNA/RNA Kit (TransGen Biotech, China). The quadruplex real time quantitative PCR method and reported methods were used to simultaneously detect the clinical samples (Wan et al., 2018a; Wan et al., 2018b; Wan et al., 2019a; Zhang et al., 2021). The results of these methods were assessed for agreement with the quadruplex real time quantitative PCR method.

## 3 Results

### 3.1 Optimisation of the quadruplex real time quantitative PCR method

Optimisation of annealing temperature, primer and probe concentration using temperature gradient PCR method and single control variable method. Smaller Ct values, smooth amplification curves and higher fluorescence signals were chosen as the optimal reaction conditions. The quadruplex real time quantitative PCR reaction system and reaction procedures were optimised using different annealing temperatures (59°C-63°C), primers and probe concentrations (0.1-1 µM). The results described that when the primers and probes concentrations of MDPV, GPV, DuCV, and DAdV-3 were 0.3 and 0.2 μM, 0.4 and 0.3μM, 0.2 and 0.3μM, 0.25 and 0.2μM, respectively, and the annealing temperature was 60°C, the Ct values were smaller, the fluorescence signals were stronger and the amplification curves were more typical.

### 3.2 Standard curve creation

**Figure 1:**
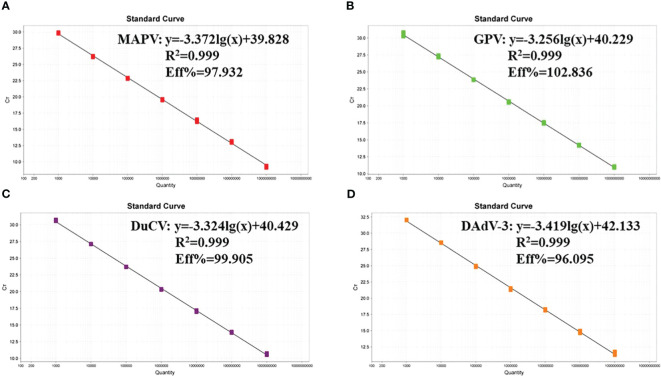
The standard curves of the quadruplex real time quantitative PCR method. **(A-D)**: Standard curves of the standard plasmid p-MAPV **(A)**, p-GPV **(B)**, p-DuCV **(C)**, and p-DAdV-3 **(D)** at final concentrations ranging from 1.0 × 10^9^ to 1.0 × 10^3^ copies/µL.

The recombinant plasmid standards of p-MAPV, p-GPV, p-DuCV, and p-DAdV-3 were mixed (1:1:1:1), ranging from 1.0 × 10^9^ to 1.0 × 10^3^ copies/µl (Three replicates of each gradient), were used to develop standard curves. The developed standard curve displayed excellent linear relationship (R^2^≥0.999) and the method was effective (**Figure 1**). MAPV, R^2^ = 0.999, Eff% = 97.932; GPV, R^2^ = 0.999, Eff% = 102.836; DuCV, R^2^ = 0.999, Eff% = 99.905; DAdV-3, R^2^ = 0.999, Eff% = 96.095.

### 3.3 Specificity analysis

**Figure 2:**
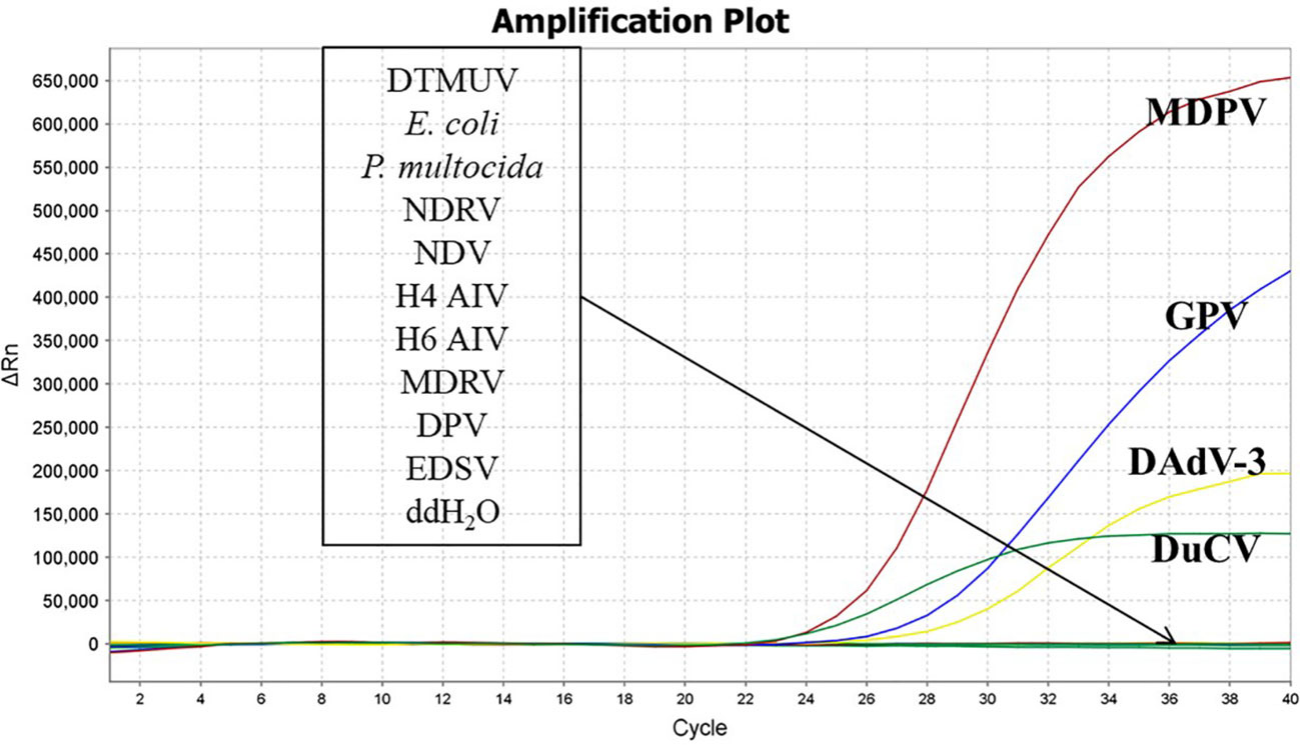
Specificity validation of the quadruplex real time quantitative PCR method.

The nucleic acids of DTMUV, *E. coli*, *P. multocida*, NDRV, NDV, H4 AIV, H6 AIV, MDRV, DPV, EDSV were used as templates. The DNA of MDPV, GPV, DuCV, DAdV-3 were used as positive controls, and ddH_2_O were used as negative controls. The results depicted that MDPV, GPV, DuCV, and DAdV-3, in accordance with the, FAM, Cy5, TAMRA and VIC fluorescence channels, generated amplification curves, while other pathogens and ddH_2_O did not appeared amplification curves. The results depicted that our established method was highly specific. (**Figure 2**).

### 3.4 Sensitivity and repeatability analysis

**Figure 3:**
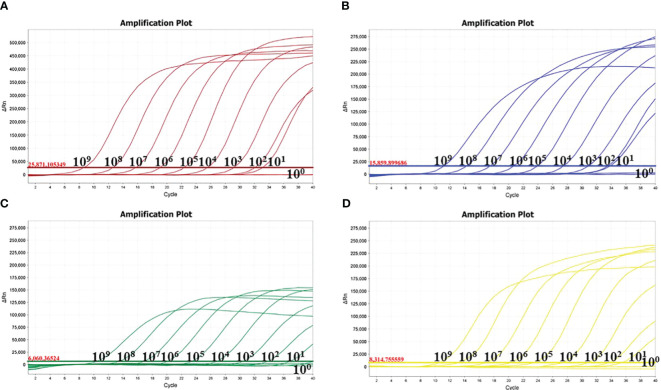
Sensitivity of the quadruplex real time quantitative PCR method. The amplification curves were generated by using the recombinant plasmid standards p-MAPV **(A)**, p-GPV **(B)**, p-DuCV **(C)**, and p-DAdV-3 **(D)** 1-10: 1.0 × 10 9 -1.0 × 10 0 copies/µL (final concentration).

The sensitivity results depicted that the minimum detection limits were 10 copies/μl for MDPV, GPV, and DAdV-3, and 1 copy/μl for DuCV (**Figure 3**). MDPV, GPV, DucV, and DAdV-3 positive controls (FAM, Cy5, TAMRA, and VIC) all had typical S-shaped amplification curves, and negative controls (FAM, Cy5, TAMRA, and VIC) all had no amplification curves and a Ct value ≥40 or no value. The test is valid if this condition is met. If the Ct value of the test sample is <36 and a typical amplification curve appears, it is judged as positive; when 36≤Ct value <40, it is judged as suspicious and doubled for re-testing; when the Ct value is ≥40 or no value and no typical amplification curve, it is judged as negative. Repeatability test displayed that the CVs was 1% - 2% (**Table 3**), this indicates that the method has good reproducibility.

**Table 3:** Reproducibility of the quadruplex real time quantitative PCR method.

Standard plasmid	Concentration of template (copies/μL)	Intra-coefficient of variation		Inter-coefficient of variation	
		X ± SD	CV (%)	X ± SD	CV (%)
p- MAPV	10^7^	16.224 ± 0.041	0.25	16.342 ± 0.056	0.34
	10^5^	22.968 ± 0.100	1.00	22.610 ± 0.096	0.42
	10^3^	29.712 ± 0.051	0.17	29.337 ± 0.042	0.14
p- GPV	10^7^	17.315 ± 0.062	0.36	17.437 ± 0.110	0.63
	10^5^	24.010 ± 0.110	0.46	23.949 ± 0.096	0.40
	10^3^	30.461 ± 0.206	0.67	30.687 ± 0.176	0.57
p- DuCV	10^7^	17.620 ± 0.192	1.09	17.161 ± 0.253	1.47
	10^5^	23.809 ± 0.089	0.37	24.012 ± 0.103	0.43
	10^3^	30.570 ± 0.303	0.99	30.450 ± 0.284	0.93
p-DAdV-3	10^7^	18.201 ± 0.292	1.60	18.161 ± 0.153	0.84
	10^5^	25.038 ± 0.098	0.39	24.984 ± 0.113	0.45
	10^3^	31.876 ± 0.123	0.39	31.950 ± 0.254	0.79

### 3.5 Clinical sample detection

**Figure 4:**
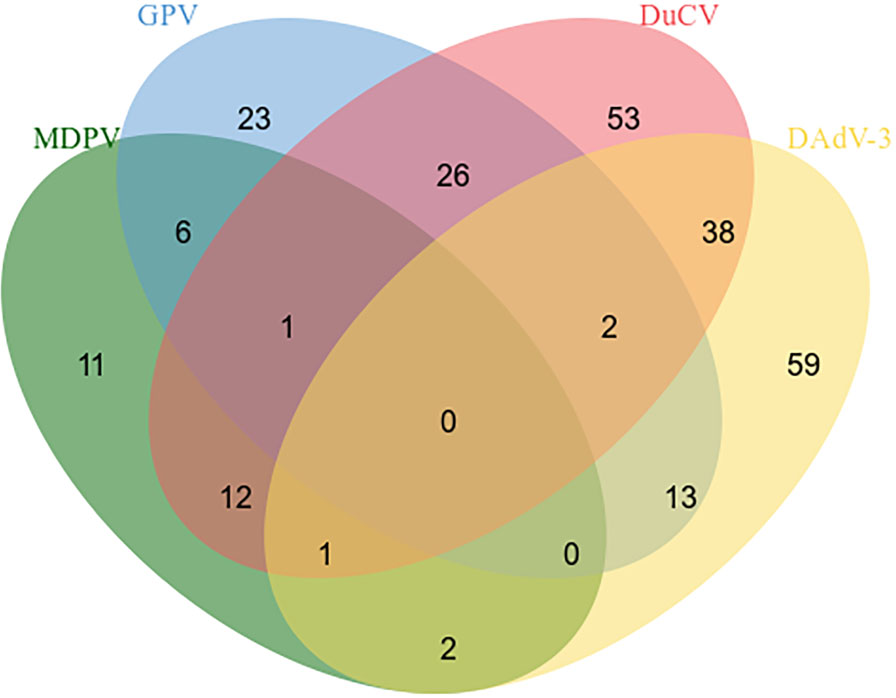
Mixed infections in positive samples.

396 clinical specimens of ducks (blood, lymph nodes, spleens, renal, intestinal, and et al) were tested by the quadruplex real time quantitative PCR method and the reported method. The results depicted that the positive rates for MDPV, GPV, DuCV, DAdV-3 were 8.33% (33/396), 17.93% (71/396), 33.58% (133/396), and 29.04% (115/396), respectively. The mixed infections of the positive samples are shown in **Figure 4**. We compare the tested samples one by one; the compliance rates were all higher than 98%, indicating that our established method is more effective in detection.

## 4 Discussion

Duck farming is an important part of the livestock industry (Luo et al., 2023). And during the rearing process, ducks are susceptible to epidemics and cause huge economic losses (Luo et al., 2023). MDPV infection has been covered in duck flocks in southern China, with mortality rates of 25-40% in 2019 (Shen et al., 2020). Wan et al. tested diseased duck samples and found 37.5% and 18.75% positive for MDPV and GPV respectively, with a mixed infection rate of 12.5% (Lin et al., 2019). Yang et al. found GPV positivity of 82.8%, DuCV positivity of 78.9% and a mixed infection rate of 70% in the hair sacs of Cherrydale ducks with deflowering syndrome (Yang et al., 2020). The 2018-2020 epidemiological survey of DAdV-3 in southern China showed that 69.23% of duck flocks were positive for the virus, with mortality rates ranging from 0.13% to 33.26% (Yin et al., 2022). The above studies show that these four viruses are highly prevalent in duck flocks, have particularly complex infections, are difficult to identify and are very damaging to the duck industry.

The present study, we developed a quadruplex real-time quantitative PCR method for the simultaneous test of MDPV, GPV, DuCV, and DAdV-3. The standard curves described good linear relationships with R^2^ greater than 0.990 and high amplification efficiencies, all between 90% and 110%. The assay was able to specifically detect MDPV, GPV, DuCV and DAdV-3 and had no cross-reactivity with 10 pathogenic bacteria susceptible to infect ducks, including DEV, EDSV, and et al. We used the MGB probe method with a minimum detection limit of 10 copies/μl for MDPV, GPV and DAdV-3 and 1 copy/μl for DuCV. The sensitivities were all higher than those previously reported for MDPV (29.7 copies/μl) (Wan et al., 2018a), GPV (50.2 copies/μl) (Wan et al., 2019b), DuCV (39.4 copies/μl) (Zhang et al., 2021) and DAdV-3 (40.9 copies/μl) (Wan et al., 2018b) by a single fluorescent quantitative PCR assay. And the coefficients of variation were less than 2% for both inter- and intra-batch testing. In addition, all four viruses are DNA viruses that can be amplified without reverse transcription, reducing the detection time. The DNA extracted from samples is more stable and less susceptible to degradation than RNA, making the test more accurate and reducing the false negative rate. The method can rapidly and accurately detect single or mixed infections in clinical specimens, facilitates early clinical detection of related diseases and enables rapid epidemiological surveys.

Critical to the accuracy of the assay is the selection of a conserved and specific target design primer. The former study showed that MDPV and GPV are also pathogenic to Muscovy ducklings (Wan et al., 2016; Wan et al., 2018a). Compared with MDPV (strain FM) and GPV (strain B), their nucleotide similarity at the genomic level is more than 80.0% (Wang et al., 2017). Furthermore, the nucleotide and amino acid identities of the two viruses at the NS gene level were 83.0% and 90.6%, respectively, and at the VP1 gene level were 81.5% and 87.6%, respectively (Wan et al., 2019a). The possibility of immune cross-reactivity between MDPV and GPV is suggested by the high degree of amino acid identity of the VP1 protein (Wang et al., 2016; Soliman et al., 2020). Therefore, differentiation between MDPV and GPV in Muscovy ducklings is essential. However, the high homology in nucleotide identities and immunogenic cross-reactivity between MDPVs and GPVs increases the risk of missed and misdiagnosed cases in the specific detection of MDPV (Dai et al., 2022). The genes that have been reported to be targeted in the MDPV genome include *Rep*, *VP3*, *VP1*, and *NS*, and GPV genome include *VP3* and *NS* (Wan et al., 2018a; Dong et al., 2019; Wan et al., 2019b; Dai et al., 2022; Liu et al., 2023). In this study, the gene sequences of MDPV and GPV were downloaded from the NCBI database (https://www.ncbi.nlm.nih.gov/), after comparison, two groups of specific primers and probes were identified on the *NS* genes of each virus and verified that no cross-reactivity occurred. DuCV-1 and DuCV-2 are widespread in China, but studies have shown that DuCV3 has been found in duck farms in Hunan, China (Liao et al., 2022). In the main reported use of the *Rep* gene for DuCV detection (Yin et al., 2023), we found that there were single base mutations when designing primers by comparing the gene, and we used concatenated bases to improve accuracy. It has been reported that the target genes used for the detection of DAdV-3 are the *Hexon* gene*, Rep* gene, and *fibre-2* gene (Wan et al., 2018b; Li et al., 2022), of which the *fibre-2* gene has great type and subgenus specificity (Yin et al., 2019), so we chose this gene as the target to establish the qPCR method.

Our application of the established quadruplex real-time quantitative PCR assay to 396 samples showed that single or mixed positivity rates for MDPV, GPV, DuCV and DAdV-3 were consistent with reported epidemiological trends for the four viruses. This suggests that multiple-pathogen co-infections at duck farms are still an important problem in duck herds. Co-infections cause more heterogeneous responses than do single infections (Zhang et al., 2015; Liu et al., 2020b). In particular, DuCV is an immunosuppressant virus that primarily affects the nutrition and growth of ducks, strikes the duck’s immune system, worsens the clinical symptoms of sick ducks, and increases the death rate of sick ducks (Yin et al., 2023). Opportunistic pathogens strike ducks and mixed and secondary infections occur when the body’s immune system is compromised (Shen et al., 2023). This is consistent with the results of our testing of clinical specimens, which have the highest rate of DuCV positivity and a higher likelihood of co-infection with other pathogens. Therefore, increased testing of duck flocks, timely removal of positive ducks, improved feed management and improved environmental hygiene are extremely important for the prevention and control of duck-related diseases.

In conclusion, we have established a sensitive, specific, rapid and efficient quadruplex real-time quantitative PCR assay for the simultaneous detection of MDPV, GPV, DuCV and DAdV-3, which provides technological reserve for the clinical prevention and control of duck diseases.

## Data Availability

The original contributions presented in the study are included in the article/supplementary material. Further inquiries can be directed to the corresponding authors.
